# Zr-Induced high temperature resistance of polymer microsphere based on double crosslinked structure

**DOI:** 10.1039/c8ra02747a

**Published:** 2018-05-30

**Authors:** Zhiyong Wang, Meiqin Lin, Meng Gu, Zhaoxia Dong, Juan Zhang, Zihao Yang

**Affiliations:** Research Institute of Enhanced Oil Recovery, China University of Petroleum Beijing 102249 China 13910509321@163.com +86-10-89734612

## Abstract

In order to obtain polymer microspheres for profile control and water shutoff with high temperature resistance and good swelling properties, micrometer microspheres with a double crosslinked structure were synthesized using the monomers acrylamide (AM), *N*-vinylpyrrolidone (NVP) and 2-acrylamide-2-methylpropanesulfonic acid (AMPS), an initiator of potassium persulfate, a crosslinking agent of *N*,*N*-methylene bis acrylamide and zirconium acetate. The crosslinked Zr–AM/NVP/AMPS microspheres were fully characterized with several means including FT-IR, ^13^C NMR, TG-DSC and SEM. Metal crosslinking was introduced into the polymer microspheres to improve the temperature resistance by crosslinking the hydrolyzed polymer molecular chains. The results of optical microscopy and scanning electron microscopy demonstrated that a double crosslinked structure (DCS) was formed inside the Zr–AM/NVP/AMPS microspheres. It is regrettable that there is no three-dimensional network structure in the microspheres with single organic crosslinked structure (SCS). In aqueous solution, the DCS polymer microspheres were able to maintain long-term thermal stability for 150 days even at a high temperature of 140 °C. The microspheres with SCS can only be preserved for 5 days in high temperature aqueous solution at 140 °C. The TGA-DSC results indicated that the aerobic temperature capability of the DCS microspheres has been greatly improved compared with HPAM and SCS microspheres. The DCS microspheres with ultra-high temperature resistance will have broad application prospects in high temperature reservoirs.

## Abstract 2

Three monomers, acrylamide (AM), *N*-vinylpyrrolidone (NVP) and 2-acrylamide-2-methylpropanesulfonic acid (AMPS), were crosslink-copolymerized in an inverse suspension system, obtaining crosslinked AM/NVP/AMPS microspheres.

## Graphical abstract

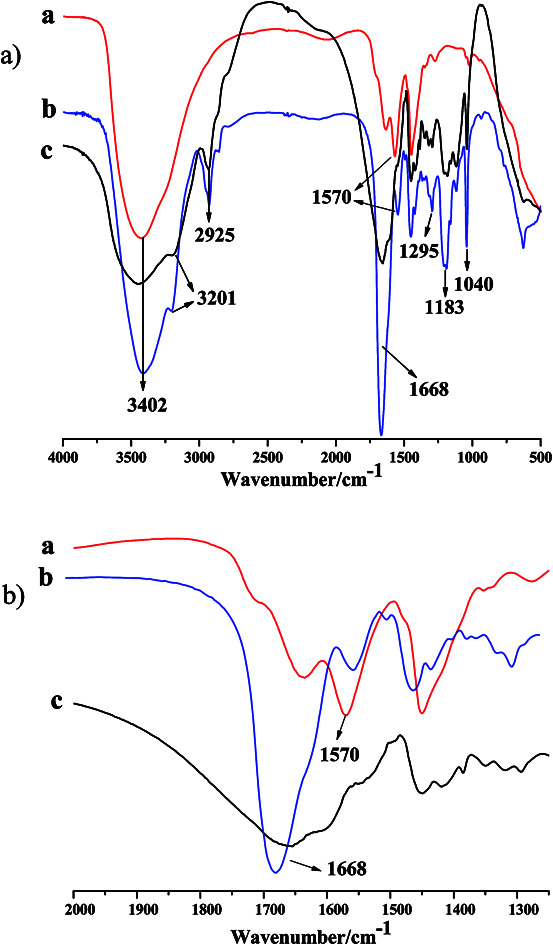

## 1. Introduction

As an important oil displacement agent in the deep profile control technique of the tertiary recovery phase, polyacrylamide (PAM) microspheres are very effective in increasing and stabilizing crude oil production.^1,2^ Because of their flexible particle size, good swelling properties, excellent elasticity and good injection properties, PAM microspheres can migrate deep into the reservoir and achieve deep profile control.^3^ Though these attractive characteristics could lead to the use of PAM microspheres in the tertiary recovery phase, the enormous challenges of good swelling properties and long-term high temperature performance still remain for the development of advanced profile control and water shutoff materials, including injectable polymer microspheres. For example, almost all of the amide groups in polymer molecular chains are hydrolyzed in just about 30 days at 100 °C, which would seriously affect the plugging performance and the displacement control performance of microspheres.^4,5^

Polymer microspheres applied in tertiary oil recovery are strongly limited by their poor temperature resistance. One of the most visible deficiencies of poor temperature resistance is that the ordinary polymer microspheres have a single organic crosslinked structure (SCS). The SCS polymer microspheres degrade rapidly at high temperature because there are very few firm cross-linked structures to resist the degradation of the polymer molecular chains. In addition, the crosslinking points are densely distributed and the crosslinked structures cannot swell very well in aqueous solution. Much effort has been devoted to improving the temperature resistance of polymer microspheres, but the feasibility still remains dissatisfactory.^6,7^ In recent years, three different kinds of method to improve the temperature resistance of polymer microspheres have been developed. One method that has a certain effect on this difficult problem, the introduction of large lateral groups (for instance, 2-acrylamide group-2-methyl propyl sulfonic acid (AMPS) and 2-acrylamide-2-phenyl ethyl sulfonate (AMSS)) into the preparation of PAM microspheres can inhibit the hydrolysis of acrylamide.^8,9^ Li^10^ synthesized AM/AMPS polymer microspheres with SCS by inverse suspension polymerization. These AM/AMPS polymer microspheres have been applied in profile control and water shutoff in the reservoir at 85 °C. Fortunately, the AM/AMPS/NVP microspheres with SCS were stable for 120 days in the environment at 90 °C.^10^ Owing to the introduction of large lateral temperature-resistant monomers, the polymer microspheres exhibit effective temperature resistance. It is believed that the certain temperature resistance and salt tolerance of the SCS microsphere resulted from its inhibition of acrylamide hydrolysis and the binding of sulfonate with salt ions. Rigid aromatic ring monomers (divinylbenzene and styrene) have also been used to prepare PAM microspheres, and can effectively improve the temperature resistance of the microspheres.^7^ Yang *et al.*^1^ selected divinylbenzene as a single organic cross-linker, sodium lauryl polyoxyethylene ether sulfate (AES) as an emulsifier, and 2′-azobis (2-methyl propionitrile) as an initiator to prepare a new kind of polymer microsphere. The results showed that the polymer microsphere with SCS had a strong deep plugging effect in reservoirs and could be used for water shutoff at 115 °C and 50 000 mg L^−1^ salinity after aging for 3 months. Similarly, water soluble phenolic resin was also used as a temperature-resistance monomer to improve the heat resistance of microspheres. They found that this new SCS polymer microsphere possessed excellent temperature resistance (140 °C, 17 days).^11^ Unfortunately, the toxicity of phenolic resin is very high, and the production conditions are harsh. These three methods mentioned above can improve the temperature resistance of polymer microspheres to some extent, but cannot satisfy the demand of high temperature and high salinity reservoirs including the Qinghai oil field,^12^ the Zhongyuan oil field,^13^ the Tarim oil field^14^ and the Tahe oil field.^15^ It can be seen that the ability of the temperature-resistance monomer to improve the heat resistance of the SCS polymer microspheres is limited, and it is necessary to find a novel crosslinking agent to improve the temperature resistance of the SCS polymer microspheres.

**Scheme 1:**
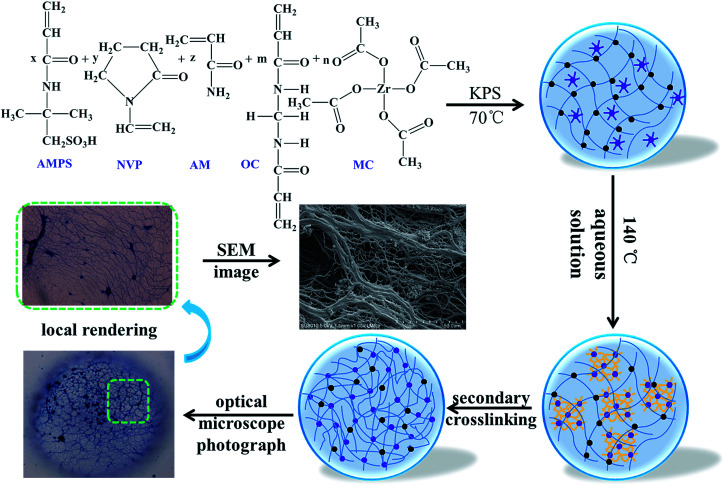
Proposed formation process of the double crosslinked structure polymer microspheres.

Metal crosslinking agents have become a polymeric material in tertiary oil recovery, and are mainly applied in the delayed crosslinking of gels and the fracturing fluid.^16,17^ Very little work has been done on incorporating kinds of metal crosslinking agents into polymeric material structures, and the improvement in temperature resistance was far higher than for the three temperature-resisting monomers mentioned above. Herein, we reported a novel high temperature-resisting microsphere with a double crosslinked structure (DCS), in which a metal crosslinking agent acted as both the crosslink and the ligand complex. The temperature resistance process and mechanism of such polymer microspheres is presented in Scheme 1. The organic cross-linker (OC) is represented by the black origin in the scheme, and firstly generates free radical polymerization with three monomers to form a linear molecular chain. The metal cross-linker (MC) is represented by the purple stars and circles in the scheme, and dissociates in high-temperature aqueous solution, giving extra coordination space. The amide groups in the polymer chains inside the microspheres are also hydrolyzed into many carboxylic acid bases (–COO–). The second cross-linking of the hydrolyzed polymer molecular chain with the central atom (Zr) can form a complex polymer. The complex polymer has a large three-dimensional network structure, which prevents the hydrolysis of the polymer molecular chain, giving it excellent temperature resistance.

## 2. Experimental

### 2.1. Materials

The materials included acrylamide (AM, Shanghai Macklin Biochemical Co. LTD, China, 99%), sorbitan monooleate (Span80, Tianjin Guangfu Fine Chemical Research Institute, China, AR), 1-vinyl-2-pyrrolidinon (NVP, Tokyo Chemical Industry Limited Company, 99%), 2-acrylamide-2-methylpropanesulfonic acid (AMPS, Shanghai Macklin Biochemical Co. LTD, 98.0%), *N*,*N*-methylene bis acrylamide (MBA, Mclean, AR), potassium persulfate (KPS, Tianjin Fuchen Chemicals, AR), methylthionine chloride (Shanghai Macklin Biochemical Co. LTD, 90.0%), partially hydrolyzed polyacrylamide (HPAM, hydrolysis degree about 25.2%), and zirconium acetate (Shanghai Macklin Biochemical Co. LTD, Zr 15–16%). All of the above materials were used without any further purification. Deionized water was filtrated through microporous cellulose membranes with a pore size of 0.22 μm. Nuclear pore membranes with 0.4 μm pores and a thickness of 10 μm were provided by the China Institute of Atomic Energy.

### 2.2. Synthesis of the polymer microspheres by inverse suspension polymerization

A known amount of Span-80 (AR) dissolved in white oil (60 g) was added to a three-necked flask and heated to 30 °C, while the monomers (18 g AM, 0.5 g AMPS, and 1.5 g NVP), 0.2 g potassium persulfate (KPS), 0.1 g *N*,*N*′-methylene bis acrylamide and 2.0 g metal crosslinking monomer were dissolved in 18 g deionized water and fully stirred until completely dissolved, and then slowly added to the three-necked flask through an isobaric addition funnel within 10 minutes. The two phases formed a W/O micro-emulsion. The temperature was increased from 30 °C to 70 °C and maintained for 2.5 h to obtain a double cross-linked polymer microspheres micro-emulsion. The micro-emulsion was added into ethanol at a 1 : 8 ratio by volume. After adequate stirring was performed to break the micro-emulsion, copious white flocculation-like precipitate was generated at the bottom of the container. After leaching the system, the obtained filter cake was washed with ethanol at a 1 : 5 ratio by volume, and leached again. Afterward, the filter cake was dried under vacuum at 50 °C for 24 h. The treated double crosslinked polymer microspheres appeared as a white powder.

A series of other DCS and SCS microspheres were also synthesized similarly following the above procedure but changing the reaction parameters, including the stirring rate, the ratio of organic cross-linker (OC) and metal cross-linker (MC), the monomer loading, the initiator concentration, and the total amount of crosslinking agents (TAC).

### 2.3. Characterization

Fourier transformation infrared (FT-IR) spectra of the polymer microspheres were measured with a Bio-Rad FTS-6000 spectrometer.

^13^C NMR spectroscopy was performed at 25 °C on a Bruker-500 ^13^C NMR system operating at 9.4 T with corresponding ^13^C resonance frequencies of 100.6 MHz using a 5 mm one NMR™ probe.

Aerobic temperature resistance of the microspheres was essential for the persistence of profile control and water shutoff. At a heating rate of 10 °C per minute, the temperature performance of the polymer microspheres and hydrolyzed polyacrylamide (HPAM) was measured in an oxygen environment by a thermogravimetric analyzer. A NETZSCH STA409PC thermogravimetric analyzer was purchased from NETZSCH Instruments Manufacturing Co. Ltd, Germany.

One drop of a sample of the swelling microspheres was placed onto the surface of a clean glass sheet for observation under an Olympus BX41 microscope manufactured by the Olympus Company (Japan).

The solid powder of the DCS polymer microspheres was directly placed onto an electrically conductive film, the sample was sprayed with metal for 3 min and the morphologies of the DCS and SCS microspheres were observed with a SEM SU8010 manufactured by the Hitachi Company of Japan.

The size distribution of the swollen microspheres and the solid powder was investigated with a Microtrac-S3500 laser particle size analyzer produced by the Microtrac Company (USA) based on laser diffraction. This analysis was performed under a constant temperature (25 °C), and with extended Blue Wave.

### 2.4. Temperature resistance and swelling properties of the polymer microspheres

About 0.2 g of microspheres was placed in an ampoule bottle containing 40 ml deionized water, then the ampoule bottle was placed under vacuum and sealed. Finally, the sample was placed in a constant temperature environment (120 °C, 130 °C, and 140 °C), and then the swelling phenomenon was observed by an optical microscope, along with the measurement of the particle size distribution by a laser particle sizer. When the microspheres were almost completely degraded, the process of temperature resistance was considered finished.

## 3. Results and discussion

### 3.1. Fourier transformation infrared (FT-IR) spectra

**Fig. 1:**
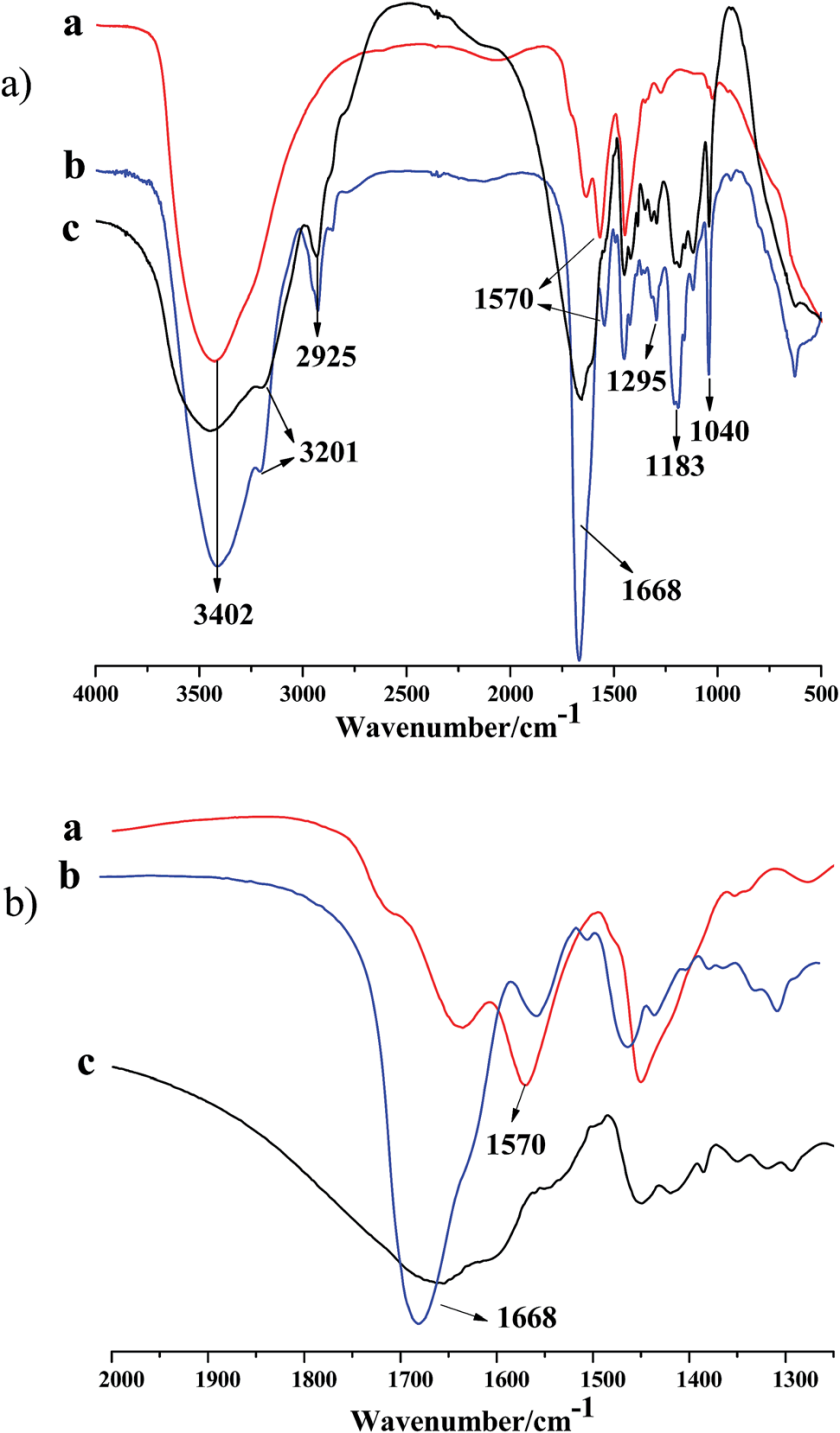
FTIR spectrum of zirconium acetate and two kinds of crosslinked microspheres (a, zirconium acetate; b, DCS microsphere; c, SCS microsphere); (a), complete diagram; (b), partial enlarged detail.

FT-IR was first utilized to confirm the successful incorporation of the functional comonomers (NVP and AMPS) into the obtained polyacrylamide microspheres. Fig. 1(a) and (b) present spectra of the metal cross-linking agent, the DCS microspheres and the SCS microspheres. In the spectra of Fig. 1(a) b and c, the characteristic absorption bands of the three kinds of monomer unit, AM, NVP and AMPS, are displayed. The bands at 1668 cm^−1^ may be attributed to the characteristic absorption of the C=O bond of the carbonyl groups in the AM, NVP and AMPS units; the bands at 1295 cm^−1^ are attributed to the characteristic absorption of the C–N bond of the amido group in the NVP unit; the bands at 3402, 3201 and 2925 cm^−1^ are ascribed to the characteristic absorption of the R_2_N–H bond of the secondary amine, the RN–H_2_ bond of the primary amine and the C–H bond of the methyl in the AM unit, respectively; the bands at 1183 and 1040 cm^−1^ correspond to the characteristic absorption of the C–N bond of the amido groups and the S=O bond of –SO_3_H in the AMPS unit. The characteristic absorption band of the double bonds of the vinyl groups (CH_2_=CH–) were covered up in the spectrum of the polymer microspheres (as shown in Fig. 1(b) b and c). In the metal crosslinking agent (Fig. 1(a) a), the band at 3402 cm^−1^ and the band at 1570 cm^−1^ represent the characteristic adsorption of hydrone and the –C–O–Zr bond of the ester group, respectively. Fortunately, the same characteristic peak (1570 cm^−1^) was found in the spectrum of the DCS microspheres, and it was not found in the spectrum of the SCS microspheres (as shown in Fig. 1(b) b and c). The above results show that free-radical polymerization of the monomers has occurred, and the Zr–AM/AMPS/NVP polymer microspheres have been formed.

### 3.2. ^13^C NMR spectroscopy

**Fig. 2:**
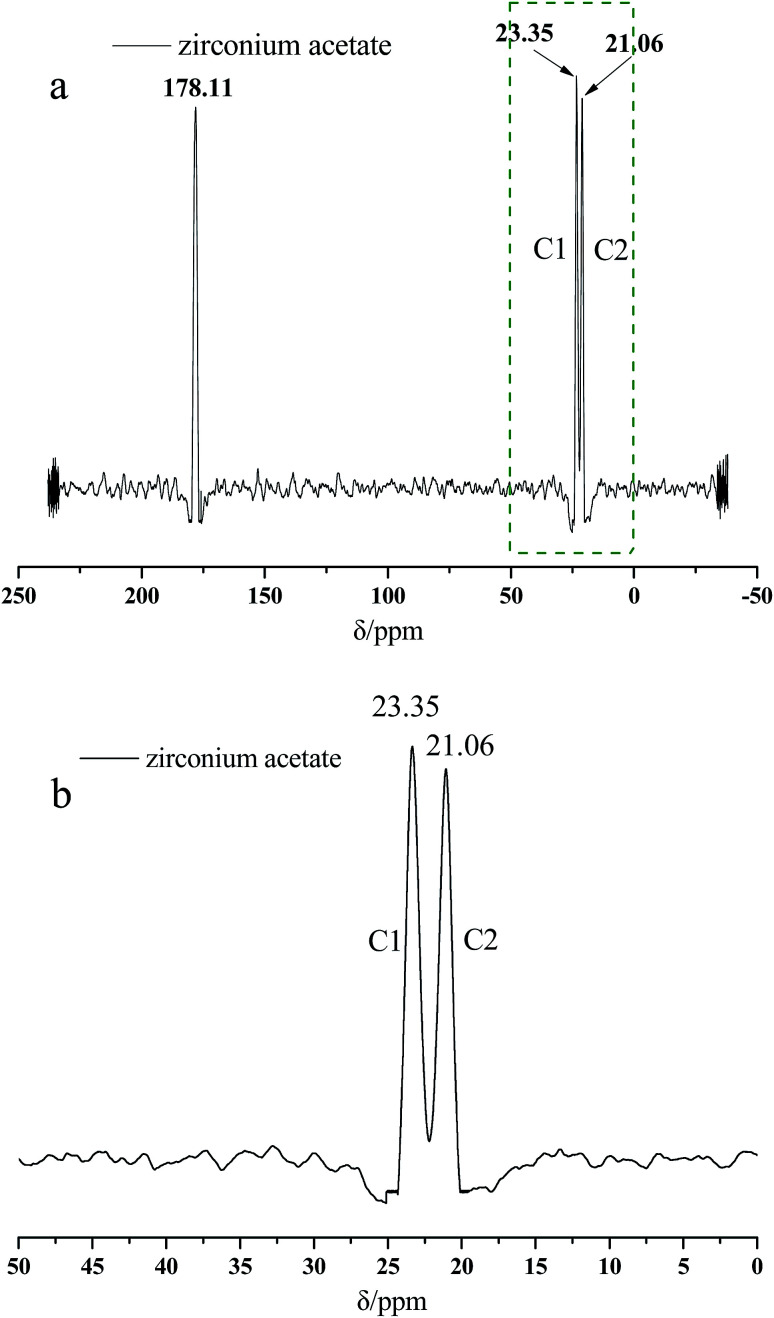
^13^C NMR Spectra of zirconium acetate (a) complete diagram; (b) partial enlarged detail.

**Fig. 3:**
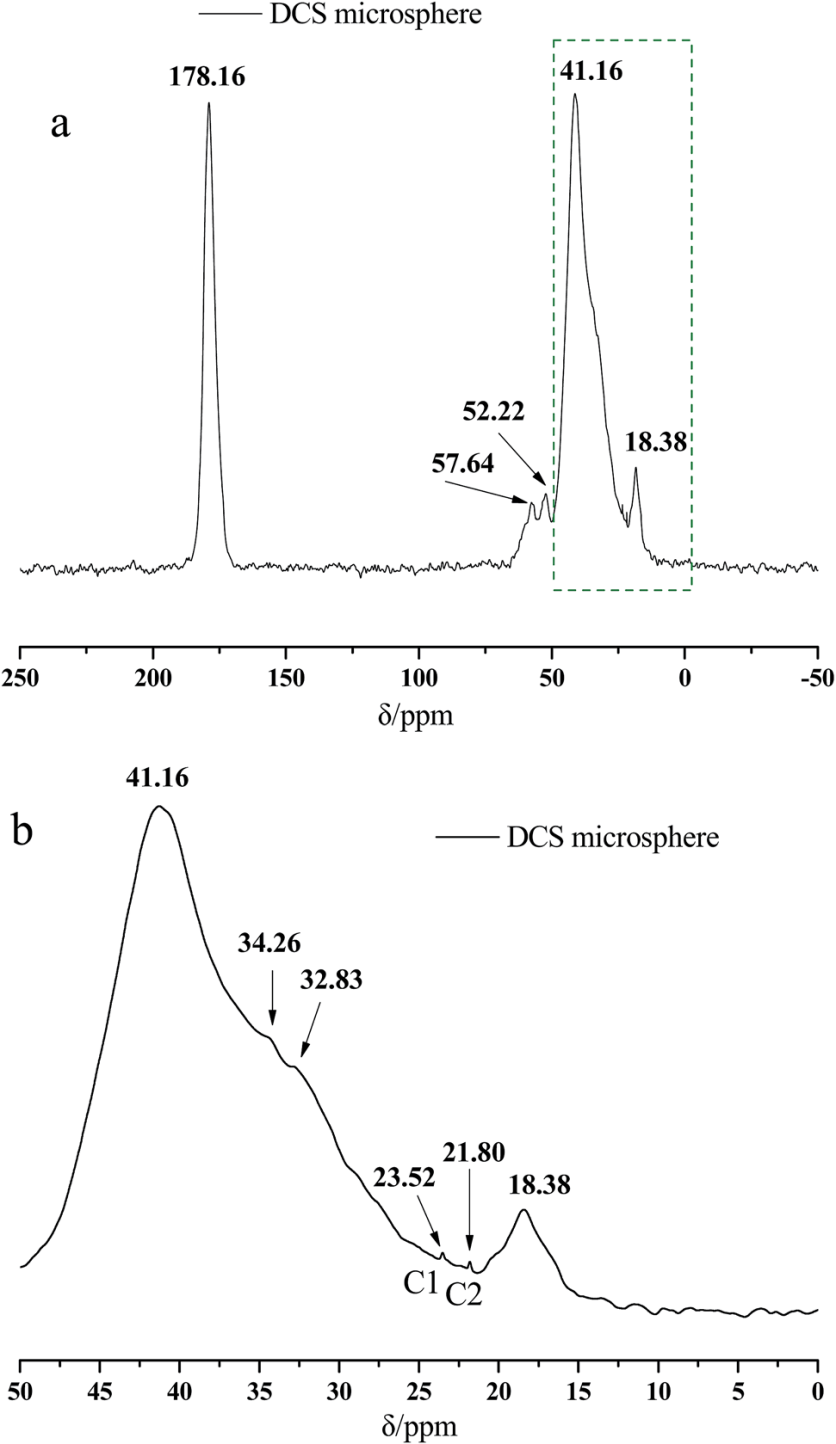
^13^C NMR Spectra of the DCS microsphere (a) complete diagram; (b) partial enlarged detail.

**Fig. 4:**
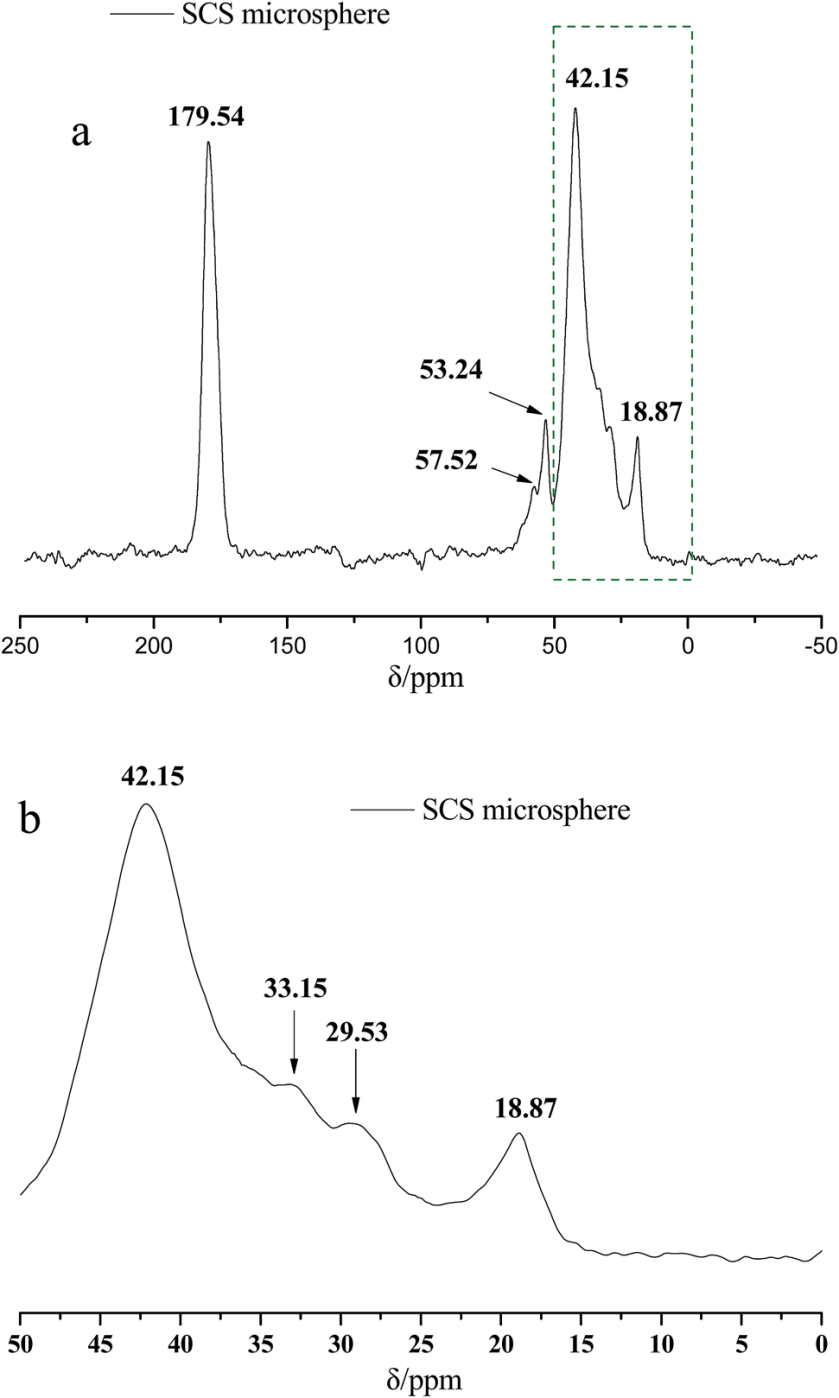
^13^C NMR Spectra of the SCS microsphere (a) complete diagram; (b) partial enlarged detail.

Fig. 2 presents the ^13^C NMR curves of the metal crosslinking agent. Deconvolution of the signals of Zr–bound acetate actually revealed two peaks (chemical shift 23.35 ppm and 21.06 ppm), of which those denoted as C1 and C2 in Fig. 2 are attributed to at least one, or two, intermediate species (Zr–acetate complex). The signal at 178.11 ppm was best fitted with one peak, and was attributed to the C=O bond of the carbonyl group. In order to better validate the existence state of metal crosslinking agent, ^13^C NMR of the DCS microspheres and the SCS microspheres was performed, shown in Fig. 3 and 4. Chemical shifts *δ* ≈ 179 ppm, 57 ppm, 53 ppm, 42 ppm, 33 ppm and 18 ppm appeared in Fig. 3 and 4. It is clear that the absorption peak of chemical shift *δ* ≈ 179 ppm should belong to the carbon atom in the C=O group. The signals at 58 ppm and 29 ppm were best fitted with two peaks, and are attributed to the sulfonic acid group (–C–SO_3_H) and the methyl group (–CH_3_) in the AMPS unit. The signals at 42 ppm, 18 ppm and 33 ppm were best fitted with three peaks, and are attributed to the three methylene groups (–CH_2_–) of the adjacent nitrogen atoms in the five membered ring of the NVP monomer. However, the signals at 23.52 ppm and 21.80 ppm appear in the ^13^C NMR of the DCS microspheres (Fig. 3), but do not appear in the ^13^C NMR of the SCS microspheres (Fig. 4). This reveals that the structure of the metal crosslinking has not been changed, and the zirconium acetate is dispersed only inside the DCS microspheres. The above results show that free-radical polymerization of the monomers has occurred, and the AM/AMPS/NVP polymer microspheres have been formed.

### 3.3. TGA-DSC of the polymer microspheres

The aerobic temperature resistance of the polymer microspheres is essential for profile control and water shutoff in high temperature reservoirs. Thermogravimetric analysis (TGA) was used to obtain information about the decomposition behavior of the polymer microspheres when heated in oxygen. According to the differential scanning calorimetry (DSC) results, the aerobic temperature capability of the polymer microspheres is characterized by a temperature point named the glass transition temperature (*T*_g_), and at this point the distance between the maximum endothermic peak and the maximum exothermic peak is equal.^18^ Determination of *T*_g_ is important for the practical use of polymer microspheres materials due to the change in characteristics triggered by the transition, thus the polymer microspheres change from an elastic to a plastic state. At temperatures above *T*_g_, the polymer microspheres had rubbery behavior, and below *T*_g_ the polymer microspheres were described as having a glass state. The rubbery state of the polymer microspheres may be described as the situation in which entanglements restrict the motion of the polymer chains, if this is resolved the polymer microspheres will behave like viscous fluid.

**Fig. 5:**
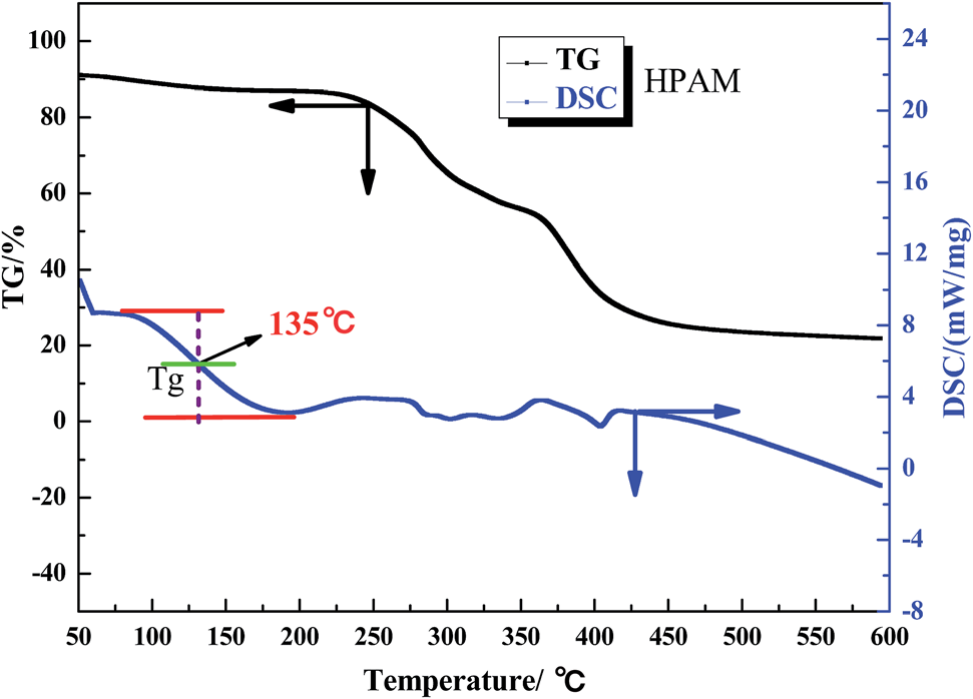
TGA and DSC results for HPAM.

**Fig. 6:**
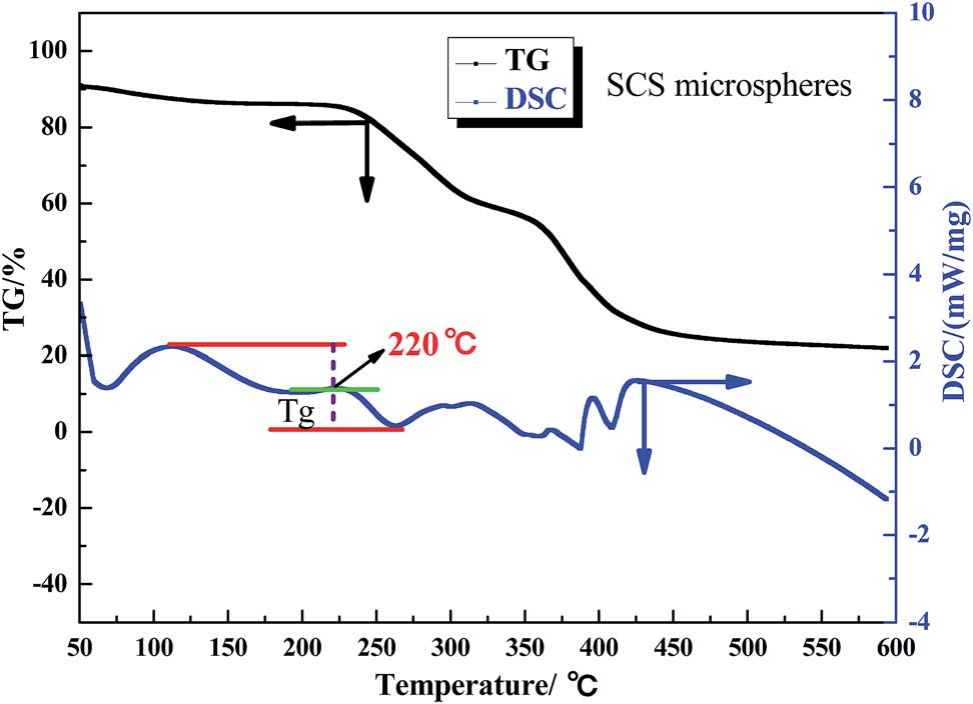
TGA and DSC results for the SCS microspheres.

**Fig. 7:**
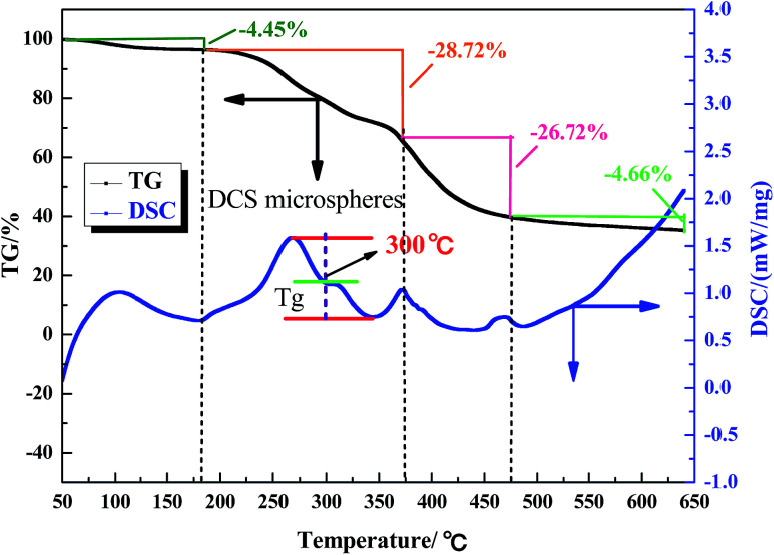
TGA and DSC results for the DCS microspheres.

**Fig. 8:**
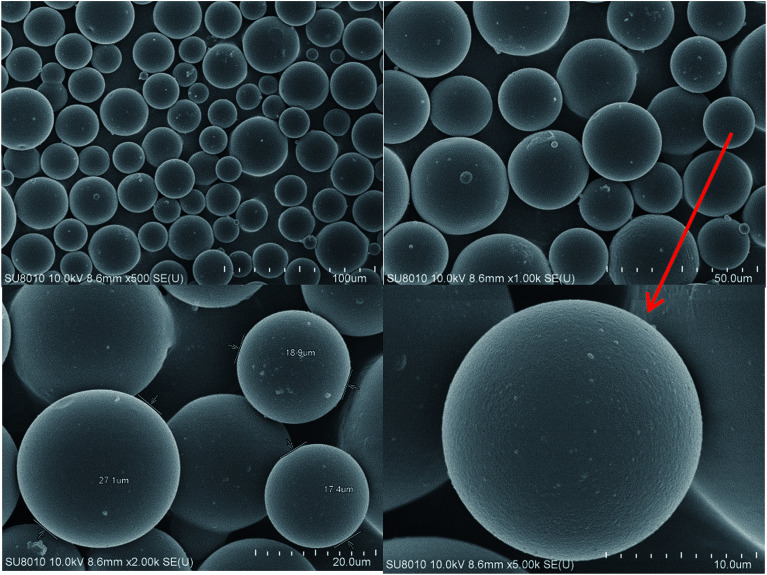
SEM photos of the dry DCS polymer microspheres at different magnification.

Fig. 5, 6 and 7 show that the aerobic temperature capability (*T*_g_) of HPAM, the SCS microspheres and the DCS microspheres was 135 °C, 220 °C and 300 °C, respectively. As shown in Fig. 7, the weight loss of the DCS microspheres mainly occurred in three phases, which are from 50 °C to 180 °C, from 180 °C to 375 °C, and from 375 °C to 475 °C. In the first phase, the proportion of weight loss was not too much, and the weight loss (4.45%) of the DCS microspheres was mainly caused by the evaporation of bound water and the presence of a small amount of oligomers on the surface of the DCS microspheres, as shown in Fig. 8. A small amount of oligomers were longer than the length of the critical precipitation chain, so were unable to closely crosslink with the DCS microspheres. The oligomer was formed by the precipitation and aggregation of vinyl monomers, so the temperature resistance of the oligomer was very poor. However, in the second and third stages, the weight loss process had a wide temperature range, from 180 °C to 475 °C. Overall, about 55.54% weight loss of the DCS microspheres occurred, mainly in the second (28.72%) and third (26.72%) phases. The DCS microspheres were synthesized by free radical polymerization. During the formation of the DCS microspheres, they first underwent the stage of forming a nucleation and then came together into a ball. In the second phase, the molecular chains on the surface of the microspheres began to crack slowly as the temperature increased, the weight loss of the microspheres between the surface and the core is about 180–375 °C. The aerobic temperature capability of the nucleation is the strongest and the weight loss of the microsphere nucleus is about 375–475 °C due to the nucleus being mainly formed by the aggregation of oligomers, which contain more structural units of polymeric species (Zr–AM/NVP/AMPS) and exhibited outstanding aerobic temperature resistance. The SCS microspheres are mainly composed of structural units of polymeric molecular chains (AM/NVP/AMPS). The polymeric molecular chains were formed by the aggregation of oligomers, which are mainly composed of structural units of monomers and the OC, and exhibited poor aerobic temperature resistance. HPAM is a kind of polymer with a linear structure, and its aerobic temperature resistance is the worst. The aerobic temperature capability of the DCS microspheres was higher than the profile control and water shutoff of conventional HPAM above 165 °C. It can be seen that the DCS microspheres have excellent aerobic temperature resistance.

### 3.4. Temperature resistance of the polymer microspheres

**Fig. 9:**
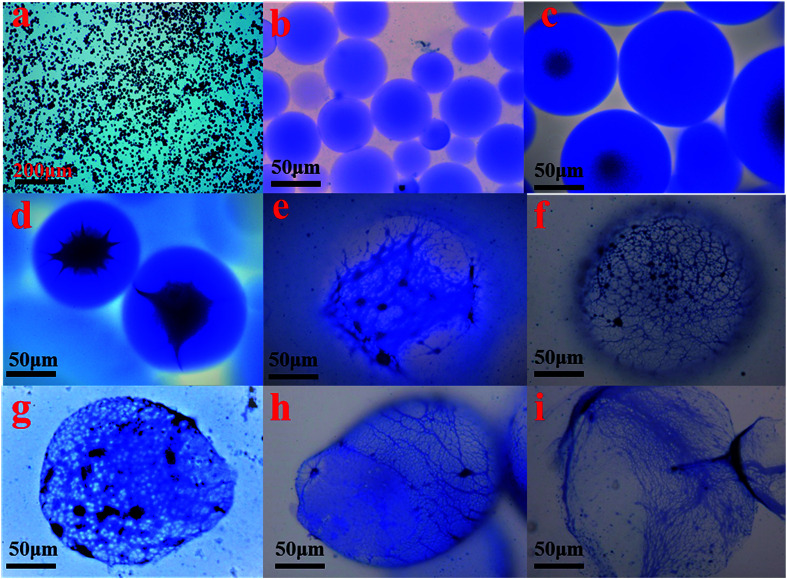
Micrograph of the DCS polymer microspheres at different swelling times at 140 °C (a) 0 days; (b) 10 days; (c) 50 days; (d) 70 days; (e) 90 days; (f) 120 days; (g) 140 days; (h) 150 days; (i) 160 days.

**Fig. 10:**
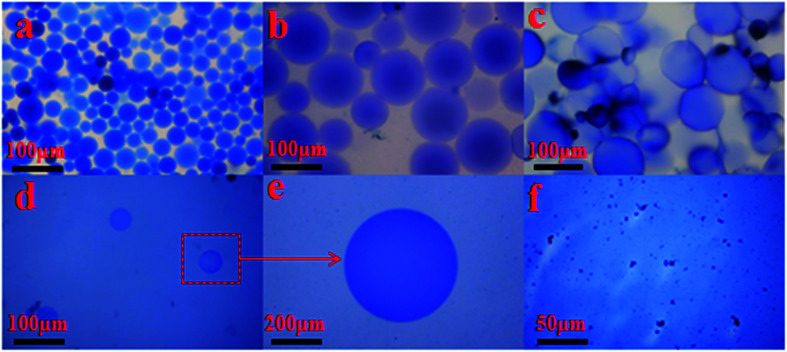
Micrograph of the SCS polymer microspheres at different swelling times at 140 °C (a) 1 day; (b) 2 days; (c) 3 days; (d) 4 days; (e) partial enlarged detail; (f) 5 days.

**Fig. 11:**
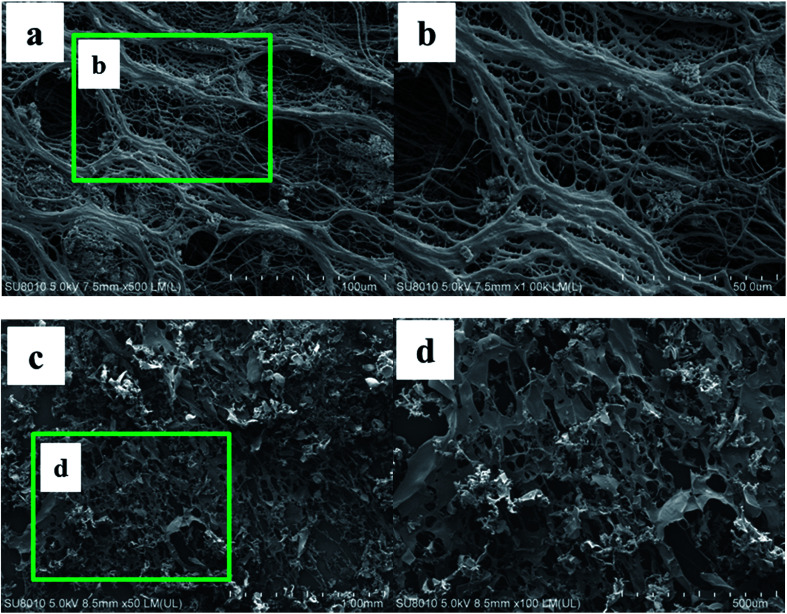
SEM images at different magnification of the swelling DCS microspheres (a and b) at 140 °C for 150 days and the swelling SCS polymer microspheres (c and d) at 140 °C for 5 days.

The formation of the double crosslinked structure in the polymer microspheres (DCS (B1)) was confirmed with an optical microscope as shown in Fig. 9. A smooth surface and a perfect spherical shape (Fig. 9b) were observed in the DCS microspheres after swelling for 10 days at 140 °C. Dark spots appeared in the center of the microspheres after 50 days (Fig. 9c). However, the structure of the microspheres obviously changed when the swelling time reached 70 days. As shown in Fig. 9d, a structure of spines appeared in the microspheres. So far, it was difficult to explain the phenomenon of the resulting polymer microspheres within the structure of spines. However, it is very likely that poly-nuclear olation complex ions were formed between the hydrolyzed MC. Fortunately, a dense network structure appeared after 90 days, as shown in Fig. 9e. Finally, a dense three-dimensional network structure filled the interior of the microsphere (Fig. 9f). With the hydrolysis of the polyacrylamide molecular chain, the structure of the acrylic acid and the poly-nuclear olation complex ions formed a more compact network structure by a coordination substitution reaction. In contrast, these phenomena did not appear in the SCS polymer microspheres (Table 1, SCS) as shown in Fig. 10. The key difference between the DCS microsphere and the SCS microsphere is the crosslinked structure existing in the DCS microsphere. The results of the current study may give a reasonable explanation. A covalent bond of the organic cross-linker and a coordination bond of the metal cross-linker were introduced into the DCS polymer microsphere at the same time. The former exerted a cross-linking effect to make the microsphere more compact, and the latter exhibited outstanding temperature resistance. With increasing swelling time, the molecular chains of the polymer microspheres began to hydrolyze, resulting in carboxylate anions (–COO^−^), which could replace the complexing water in the metal cross-linker.^19^ With the extension of time, the second crosslinking process of the MC and the poly-nuclear olation complex ions finished, accompanied with the formation of an irregular sphere (Fig. 9g). The surface of the microspheres partially burst (Fig. 9h) after 150 days and was almost broken completely (Fig. 9i) after a further 10 days. In the case of this polymeric microsphere, the double crosslinked structure (DCS) appeared when the MC underwent a ligand substitution reaction, and a dense three-dimensional network structure with double crosslinking was observed in the SEM images as shown in Fig. 11a and b. Compared with this, the swollen and broken SCS microspheres have no three-dimensional network structure, but some irregular slices, as shown in Fig. 11c and d. The reason for this is that the molecular chain formed between the monomers and the OC was used as a crosslinking point firstly, then the molecular chains of the DCS microspheres began to hydrolyze and the ligand substitution reaction mentioned above occurred, so a dense three-dimensional network structure with the metal cross-linker as the center formed in the microsphere. Obviously, the DCS polymer microspheres in aqueous solution were able to keep long-term thermal stability even at a high temperature of 140 °C. In particular, the temperature resistance of the SCS microspheres was fairly poor when compared with the DCS microspheres as shown in Table 1.

**Table 1:** The composition of the raw materials for the SCS and DCS polymer microspheres syntheses, and the properties of the microspheres

Microsphere	Synthesis					Performance testing	
	Stirring speed [rpm]	KPS [g]	AM/AMPS/NVP [wt%]	OC/MC [wt%]	TAC^a^ [wt%]	Temperature resistance (120 °C) [days]	Swelling property [times]
SCS	300	0.2	16/1.5/2.5	0.4/0	2.0	20	10.34
DCS(A1)	300	0.2	16/0/4.0	0.1/0.3	2.0	68	8.22
DCS(A2)	300	0.2	16/0.5/3.5	0.1/0.3	2.0	81	9.45
DCS(A3)	300	0.2	16/1.5/2.5	0.1/0.3	2.0	220	11.37
DCS(A4)	300	0.2	16/2.0/2.0	0.1/0.3	2.0	136	12.56
DCS(A5)	300	0.2	16/3.0/1.0	0.1/0.3	2.0	99	12.94
DCS(B1)	300	0.2	16/1.5/2.5	0.1/0.2	2.0	74	11.48
DCS(B2)	300	0.2	16/1.5/2.5	0.1/0.1	2.0	42	19.25
DCS(C1)	300	0.2	18/0.5/1.5	0.1/2.0	2.0	210	13.68
DCS(C2)	300	0.2	18/1.0/1.0	0.1/2.0	2.0	131	13.83
DCS(C3)	300	0.2	18/1.5/0.5	0.1/2.0	2.0	100	14.21
DCS(D1)	200	0.2	18/0.5/1.5	0.1/2.0	2.0	207	13.54
DCS(D2)	400	0.2	18/0.5/1.5	0.1/2.0	2.0	204	13.86
DCS(E1)	300	0.05	18/0.5/1.5	0.1/2.0	2.0	204	13.71
DCS(E2)	300	0.1	18/0.5/1.5	0.1/2.0	2.0	208	13.55
DCS(E3)	300	0.3	18/0.5/1.5	0.1/2.0	2.0	206	12.97
DCS(F1)	300	0.1	18/0.5/1.5	0.1/2.0	1.0	200	13.44
DCS(F2)	300	0.1	18/0.5/1.5	0.1/2.0	0.5	53	14.21

a The weight of the crosslinking agent is the percentage of the total mass of the monomers.

### 3.5. Swelling properties of the polymer microspheres

**Fig. 12:**
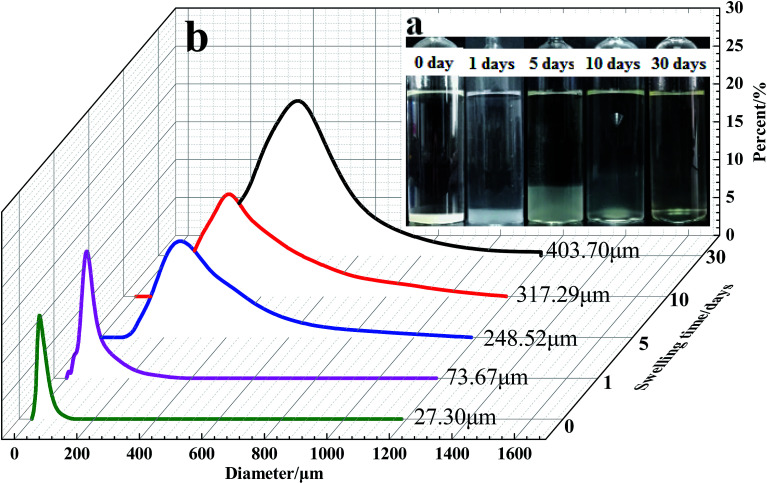
Swelling process and size distribution of the DCS microspheres with different swelling times at 140 °C (a) swelling process of the microspheres in an ampoule bottle; (b) particle size distribution of the microspheres.

**Fig. 13:**
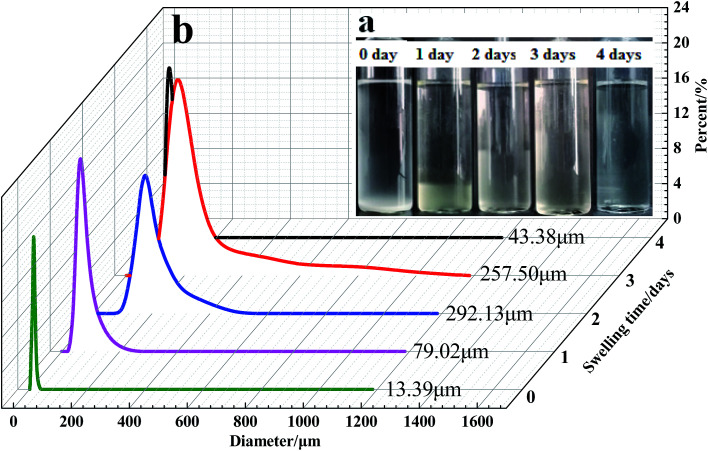
Swelling process and size distribution of the SCS microspheres with different swelling times at 140 °C (a) swelling process of the microspheres in an ampoule bottle; (b) particle size distribution of the microspheres.

The particle size distribution curves of the microspheres at different swelling times are presented in Fig. 12 and 13. According to Flory–Huggins theory, for the polymeric microspheres solution, the expansion degree of the microspheres will increase when the ionic charge density of the polymer molecules is increased or the crosslinking density and the ionic strength of the solution are decreased. As shown in Fig. 12a, the swelling time has a great influence on the swelling rate of the DCS microspheres. The average particle size of the swelling microsphere at 0, 1, 5, 10, and 30 days was 27.30 μm, 73.67 μm, 248.52 μm, 317.29 μm and 403.7 μm, respectively. The average particle size of the swelling microsphere at 30 days was 5.48 times bigger than that of the swelling microsphere after 1 day (Fig. 12b). When the swelling time increased, the average particle size gradually increased, and the entire swelling process lasted about 30 days, as shown in Fig. 12a. It can be seen that the DCS microspheres have very prominent swelling properties. However, the swelling process of the SCS microspheres lasted about 3 days at 140 °C (Fig. 13a). It is obvious that the average particle size (43.38 μm) of the swelling SCS microspheres at 4 days showed a significant decrease, as shown in Fig. 13b. The polymer microspheres possessed a three-dimensional network structure with many interlinked points, and the intertwined polymer chains are well distributed within the microspheres, which could easily swell in the high temperature aqueous solution. With an increase in swelling time, the twisted polymer chains will gradually straighten until the entire molecular chain of the polymer particles is fully straight and the swelling process is over. The structure of the single organic crosslinked network quickly hydrolyzed with increasing swelling time inside the SCS microspheres. With the hydrolysis of the polyacrylamide molecular chain, which had a second crosslinking interaction with the metal crosslinking agent, as a result the structure of the acrylic acid and the poly-nuclear olation complex ions formed a more compact network structure by a coordination substitution reaction inside the DCS microspheres.^20^ Obviously, the main reason for the different swelling properties of the microspheres is the difference in crosslinking structure of the DCS microspheres and the SCS microspheres.

## 4. Conclusion

Three monomers, acrylamide (AM), *N*-vinylpyrrolidone (NVP) and 2-acrylamide-2-methylpropanesulfonic acid (AMPS), were crosslink-copolymerized in an inverse suspension system, obtaining crosslinked AM/NVP/AMPS microspheres. By making use of the ligands of the AM/NVP/AMPS microspheres, coordinate bonds to a metal crosslinking agent can be introduced into the AM/NVP/AMPS microspheres *via* the reaction of these ligands with zirconium acetate, resulting in double crosslinked Zr–AM/NVP/AMPS. In order to obtain Zr-AM/NVP/AMPS microspheres with ultra-high temperature resistance, the stirring rate, the ratio of the OC and the MC, the monomer loading, the initiator concentration, and the total amount of crosslinking agent used should be effectively controlled so as to create the optimum reaction conditions. The Zr–AM/NVP/AMPS microspheres have a double crosslinked structure and have higher temperature resistance and better swelling capacity than the single organic crosslinked polymer microspheres. The aerobic temperature capability of the DCS microspheres has been greatly improved compared with HPAM and the SCS microspheres.

## Conflicts of interest

There are no conflicts to declare.

## Supplementary Material
